# Magnetic Silver Nanoparticles Stabilized by Superhydrophilic Polymer Brushes with Exceptional Kinetics and Catalysis

**DOI:** 10.3390/polym16172500

**Published:** 2024-09-02

**Authors:** Asghar Dolatkhah, Chandni Dewani, Masoud Kazem-Rostami, Lee D. Wilson

**Affiliations:** 1 Department of Chemistry, University of Saskatchewan, 110 Science Place, Saskatoon, SK S7N 5C9, Canada; 2 Department of Chemical Engineering, Malaviya National Institute of Technology Jaipur, Jawahar Lal Nehru Marg, Jhalana Gram, Malviya Nagar, Jaipur 302017, Rajasthan, India; 3 Department of Chemistry, Northwestern University, Evanston, IL 60208, USA; masoud.kr@gmail.com; 4 Faculty of Science and Engineering, Macquarie University, North Ryde, NSW 2109, Australia

**Keywords:** chitosan, polymer brush, catalyst, water treatment, nanoparticle

## Abstract

Stimuli-responsive catalysts with exceptional kinetics and complete recoverability for efficient recyclability are essential in, for example, converting pollutants and hazardous organic compounds into less harmful chemicals. Here, we used a novel approach to stabilize silver nanoparticles (NPs) through magneto/hydro-responsive anionic polymer brushes that consist of poly (acrylic acid) (PAA) moieties at the amine functional groups of chitosan. Two types of responsive catalyst systems with variable silver loading (wt.%) of high and low (PAAgCHI/Fe_3_O_4_/Ag (H, L)) were prepared. The catalytic activity was evaluated by monitoring the reduction of organic dye compounds, 4-nitrophenol and methyl orange in the presence of NaBH_4_. The high dispersity and hydrophilic nature of the catalyst provided exceptional kinetics for dye reduction that surpassed previously reported nanocatalysts for organic dye reduction. Dynamic light scattering (DLS) measurements were carried out to study the colloidal stability of the nanocatalysts. The hybrid materials not only showed enhanced colloidal stability due to electrostatic repulsion among adjacent polymer brushes but also offered more rapid kinetics when compared with as-prepared Ag nanoparticles (AgNPs), which results from super-hydrophilicity and easy accumulation/diffusion of dye species within polymer brushes. Such remarkable kinetics, biodegradability, biocompatibility, low cost and facile magnetic recoverability of the Ag nanocatalysts reported here contribute to their ranking among the top catalyst systems reported in the literature. It was observed that the apparent catalytic rate constant for the reduction of methyl orange dye was enhanced, PAAgCHI/Fe_3_O_4_/Ag (H) ca. 35-fold and PAAgCHI/Fe_3_O_4_/Ag (L) ca. 23-fold, when compared against the as prepared AgNPs. Finally, the regeneration and recyclability of the nanocatalyst systems were studied over 15 consecutive cycles. It was demonstrated that the nanomaterials display excellent recyclability without a notable loss in catalytic activity.

## 1. Introduction

*Functional polymer brushes* with polyionic arms have been a topic of longstanding interest, as they have a large hydrodynamic volume and stimulus-specific sensitivity [1,2,3,4,5,6,7,8]. Hybrid materials composed of magnetic polymer brushes decorated with nanocatalysts (NCs) can constitute durable supports for the design and synthesis of stimuli-responsive systems [9,10,11]. Ag noble metal nanoparticles (NPs) in the last 20 years have been one of the most important and indispensable tools in the design of heterogeneous catalysts for a broad array of applications [12,13,14,15]. Among various catalyst supports, stimuli-sensitive polymer brushes have attracted the interest of researchers’ because of their tunable properties and functionality [16]. Additionally, embedding metal NPs within stimuli-sensitive polymer brushes [17] has emerged as a promising strategy to generate highly stabilized stimuli-sensitive catalytic nanoreactors. Stabilization of catalyst NPs on polymer brushes can effectively ensure long-term stability, enhanced accessibility of catalyst active sites, fast diffusion of reactants/products and tunability of catalytic activity due to the stimuli-responsive nature of templates [18]. Magnetite nanoparticles (MNPs) demonstrate promising applications in catalysis to support active metal sites due to their water dispersibility and recyclability [9,19,20]. Compared to densely compact neutral polymeric templates, in the case of polyelectrolyte brushes, the repulsive electrostatic forces originating from tethered arms and strong hydration lead to high accessibility of Ag active sites.

Stimuli-responsive polymer brushes have the advantage of entrapment and accumulation capabilities of certain guest molecules along with an environment for in situ nucleation and stabilization of metallic NPs [21,22,23,24]. Poly(acrylic acid) [25] and star-like copolymer brush [14] systems functionalized AgNPs that possess long-term stability and adjustable catalytic properties have been reported [14]. In these cases, polymer brushes have served as templates to stabilize AgNPs for the preparation of nanocatalyst supports and to accommodate various active metal NPs. Ansar et al. [26] have introduced thiolated poly(acrylic acid) functionalized Au NPs as pH-responsive and recoverable colloidal catalysts. Star-shaped polymers [27] and thermo-responsive polymer brush functionalized Pd nanocatalysts [28] have been reported as carriers for catalytic reduction of 4-nitrophenol and methylene blue dyes. Catalytic activity of colloids comprised of poly(N-isopropylacrylamide) (pNIPAM) microgels as support materials for AgNPs have been highlighted by Tzounis et al. [22]. Although these polymer brush-based nanoreactors are highly efficient for catalysis purposes, their recovery and reuse after multiple cycles present a challenge that has attracted extensive attention [14,18].

In the current work, we report a synthetic approach that uses polymer brushes containing poly anionic groups as platform anionic ligands to stabilize magnetic Ag nanocatalysts. These systems were studied for catalytic reduction of 4-nitrophenol and methyl orange dyes as model dye substrates. An underlying hypothesis is that the super hydrophilic nature of magnetite and poly (acrylic acid) side chains ensures the availability of catalyst active sites for hydrophilic species such as 4-nitrophenol by increasing its availability near the catalyst surface. In turn, the NCs reported herein are shown to display impressive reaction kinetics. Moreover, the superior catalytic reductive activity of these systems toward 4-nitrophenol and methyl orange under multiple recovery and reuse cycles demonstrates their potential as novel and functional catalysts.

## 2. Experimental Section

### 2.1. Materials

Silver nitrate (AgNO_3_) and sodium borohydride (NaBH_4_) (purity 95.0%) were supplied by BDH Chemicals, Toronto, ON, Canada. Methyl orange, sodium hydrogen carbonate (NaHCO_3_) and potassium borohydride (KBr) (purity 99.0%) were purchased from Sigma-Aldrich, Oakville, ON, Canada. 4-nitrophenol (purity 99.0%) was obtained from Alfa Aesar, Ward Hill, MA, USA. Ferrous chloride (purity ≥ 99.0%), ferric chloride (purity ≥ 99.0%), acrylic acid (99% purity) and chitosan powder with 75–85% deacetylation (CHI, 190 kDa based on viscosity) were supplied as well by Sigma-Aldrich, Oakville, ON, Canada. All materials were used as received without further purification except 4-nitrophenol, which was purified and recrystallized three times from aqueous media. The water used in this study was purified using a Milli-Q purification system (Millipore Sigma, Oakville, ON, Canada, resistance of 18.2 MΩ/cm).

### 2.2. Instruments

The morphology of the materials was characterized using TEM (HT-7700 microscope, Hitachi, Tokyo, Japan) operating at an accelerating voltage of 200 kV. The TEM samples were prepared by drop-casting of the catalyst nanocomposite dispersion in an ethanol solution onto a carbon-coated Cu film, followed by solvent evaporation at ambient temperature before imaging. The samples were dispersed in an ethanol solution using an ultrasonic bath. DLS measurements were performed at a constant room temperature of 21 °C using a Malvern Zetasizer Nano ZS instrument from Malvern Instruments Ltd., Malvern, UK. Highly dilute aqueous solutions were analyzed using disposable cuvettes with a 90° scattering angle. To remove any interfering dust particles, all samples were filtered with 0.45 μm syringe filters before analysis. UV–vis spectra were collected using a Varian Cary 6000i Scan spectrophotometer, Santa Clara, CA, USA. The diffuse reflectance infrared Fourier transform spectra (DRIFT) were recorded with a Bio-RAD FTS-40 spectrophotometer (Santa Clara, CA, USA) in reflectance mode. The samples were mixed with pure spectroscopic grade KBr, followed by co-grinding with the sample in a 1:10 sample/KBr wt. ratio in a mortar and pestle. TA Instruments, New Castle, DE, USA (TGA-Q50) performed the thermogravimetric analysis (TGA) of the samples, which were placed in aluminum pans with a heating rate of 5 °C/min under a flow of nitrogen gas (90 mL/min).

### 2.3. Synthesis of PAAgCHI Polymer Brushes and PAAgCHI/Fe_3_O_4_

The synthesis of polyacrylic acid (PAA) grafted chitosan polymer brushes, PAAgCHI, generally has much in common with a grafting from strategy, which utilized free radical polymerization of acrylic acid monomers onto active sites of CHI, in accordance with a previous report [5,6]. Herein, the preparation of polymer brush/magnetite nanocomposites [3] (PAAgCHI/Fe_3_O_4_) employed 1 g of Fe_3_O_4_ particles dispersed in 200 mL water and sonicated for 30 min at 23 °C. Separately, 0.2 g of PAA polymer brush (2 mmol/L) was dissolved in 100 mL water and stirred at 23 °C until dissolved completely. Next, the particle dispersion was degassed for 15 min under nitrogen flow before adding the polymer solution and sonicating it for 30 min under N_2_ flow at 70 °C. Then, the nanocomposite particles were separated with an external magnet and rinsed with water before drying at 23 °C for 24 h.

### 2.4. Synthesis of PAAgCHI/Fe_3_O_4_/Ag (H, L) Catalysts

**Figure 1:**
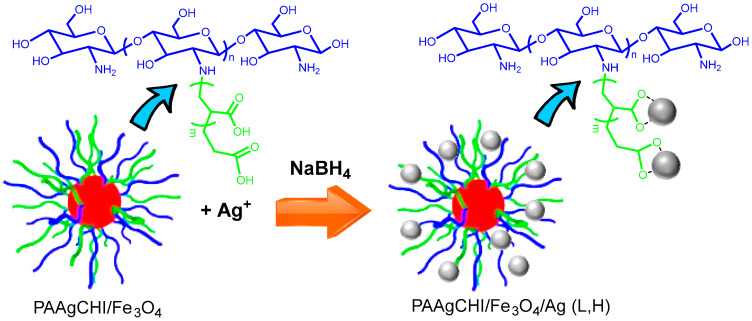
Schematic proposed structure of the stimuli-responsive catalyst. Formation of hybrid metal nanomaterial, PAAgCHI/Fe_3_O_4_/Ag (L, H). The following color scheme defines the various components: red sphere (Fe_3_O_4_), grey sphere (AgNPs), blue line (chitosan) and green line (grafted PAA; PAAg).

A mixture of 17 mg (1 mmol/L) or 8.5 mg AgNO_3_ (0.5 mol/L) for high and low content, respectively, and 50 mg of PAAgCHI/Fe_3_O_4_ nanocomposites in 100 mL Millipore water was kept stirring for 24 h, followed by a 2 h sonication the next day. A total of 300 mL of freshly prepared NaBH_4_ solution (2 mmol/L) was added dropwise to the above mixture within 55 min with sonication. The particles were cleaned with sequential magnetic separation and multiple rinsing with water before drying at 23 °C. The final products were named magnetic polymer brush-supported Ag nanocatalysts (PAAgCHI/Fe_3_O_4_/Ag (H) and PAAgCHI/Fe_3_O_4_/Ag (L), depending on the high and low silver content). AgNPs with a mean diameter of 10 nm were prepared through the reduction of AgNO_3_ with NaBH_4_ as a yellowish solution, which was centrifuged to obtain silver NPs. Figure 1 illustrates the synthetic procedure followed to produce the magnetite/polymer brush Ag nanocatalyst (NC).

### 2.5. Catalytic Activity of PAAgCHI/Fe_3_O_4_/Ag (H, L) Catalysts

The catalytic activity of the materials was examined quantitatively via the reduction of 4-nitrophenol (4-NP) to 4-aminophenol (4-AP) and reduction of methyl orange to 4-amino-benzenesulphonicacid and N,N-dimethyl-benzene-1,4-diamine as selected dyes in the presence of excess NaBH_4_ for model reactions. In a typical experiment, 5 mL of 1.1 mM 4-NP in aqueous media (pH = 8.2) was mixed with 5 mg of catalyst NC, and the mixture was sonicated 15 min in a vial. To this mixture, 10 mg of NaBH_4_ was then added, and the reaction was started immediately. Aliquots of the samples (0.2 mL) were taken at regular time intervals and diluted with water (2.8 mL). The UV–vis spectra of the solutions were recorded at regular intervals over 250–600 nm to track the reaction progress. The solutions were filtered using a 0.45 μm nylon syringe filter to yield particle-free solutions and halt the reactions prior to the UV–vis spectral measurements. The appropriate reactions were performed for reduction of methyl orange (MO) in the same manner as for 4-NP, as stated above, whereas the concentration of MO was 1 mM. This process was performed for all three catalyst samples of as-prepared AgNPs, PAAgCHI/Fe_3_O_4_/Ag (H) and PAAgCHI/Fe_3_O_4_/Ag (L). The respective rate constants of the catalysts for the dye reduction reaction were estimated by measuring the intensity of the UV–vis absorbance of 4-NP and MO with time at 400 nm and 470 nm, respectively. To study the recyclability of the PAAgCHI/Fe_3_O_4_/Ag (H) and PAAgCHI/Fe_3_O_4_/Ag (L) catalysts, the absorbance of the catalysts was measured up to 15 catalytic cycles using 10 mL of 1.1 mM 4-NP (pH = 8.2), 10 mg of catalyst and 20 mg (0.05 mol/L) of NaBH_4_. After the reaction for 20 min, the reaction mixture was kept in contact with the magnet to enable separation of the catalyst. A sample of the reacted mixture was taken, and the dye absorbance was measured using UV–vis spectrometry. The separated catalysts were washed with absolute ethanol, followed by drying in an oven for approximately 40 min after each cycle to investigate the reusability of the catalyst particles. The recovered catalyst was redispersed in a fresh solution of 4-NP for the next catalytic cycle. The *C_t_/C*_0_ ratio was calculated to examine the recyclability of the catalyst for each cycle, where *C_t_* is the concentration of 4-NP at 20 min (t = 20 min) and *C*_0_ is the concentration of 4-NP at time zero (t = 0).

## 3. Results and Discussion

With reference to Figure 1, two types of polymer-supported catalyst materials were prepared with different weight content (wt.%) of silver relative to polymer brush/magnetite nanocomposites: PAAgCHI/Fe_3_O_4_/Ag (H) and PAAgCHI/Fe_3_O_4_/Ag (L). The composite was prepared such that the polymer, PAA, could provide adsorption and immobilization sites for the silver NPs, which eventually act as the active sites for catalytic reduction. The system was supported on a magnetite nanoparticle surface for easy separation and recovery of the catalyst particles from the solution. We evaluated the successful preparation of the polymer brush and AgNPs assemblies by employing various techniques (TGA, IR, XPS, and light scattering) along with an evaluation of the various catalyst systems to evaluate the role of the polymer brush support for the catalytic reduction of 4-NP and MO dyes, respectively, as outlined below.

### 3.1. Characterization

**Figure 2:**
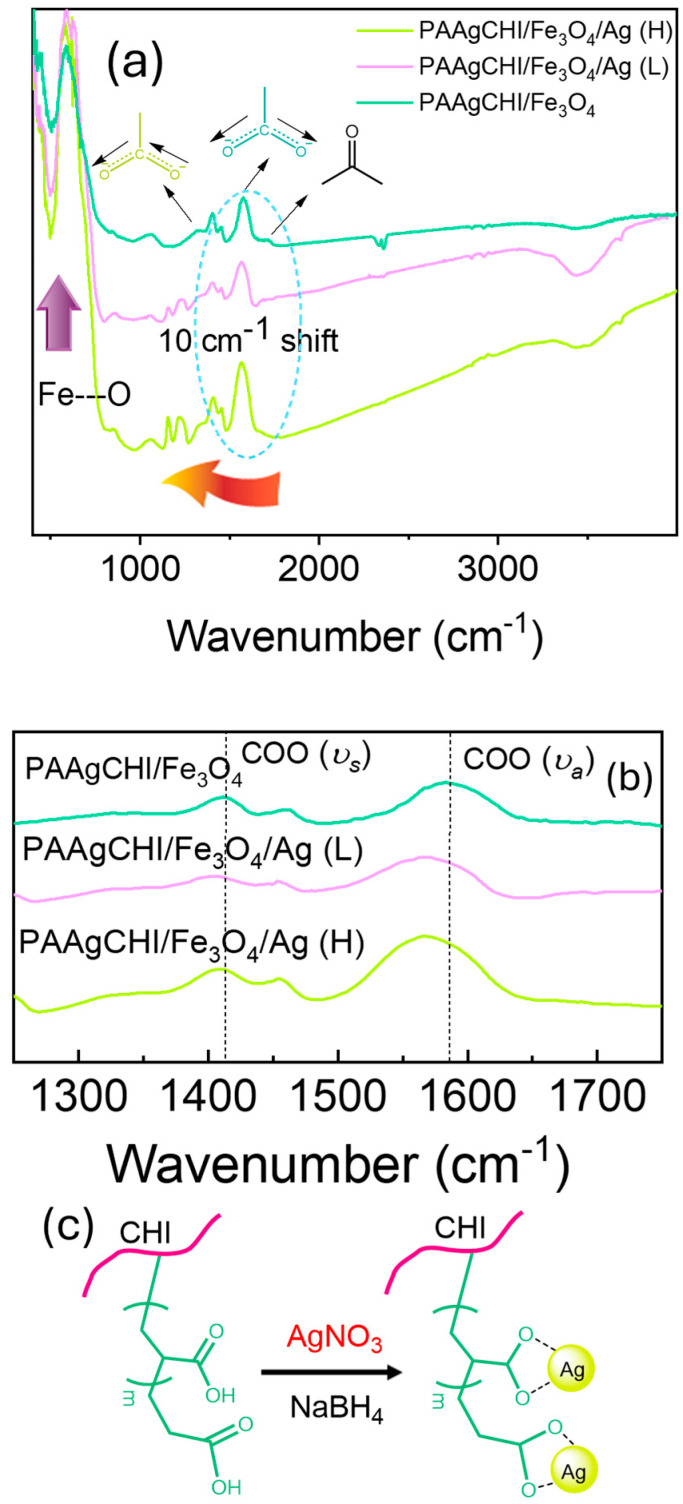
(**a**) FTIR spectra of magnetic polymer brush/Ag nanocomposites at different stages of production for PAAgCHI/Fe_3_O_4_, PAAgCHI/Fe_3_O_4_/Ag (L) and PAAgCHI/Fe_3_O_4_/Ag (H); (**b**) Expanded region of FTIR spectra showing symmetric (*υ_s_*) and asymmetric (*υ_a_*) CO_2_-stretching bands. (**c**) The corresponding chemical structure of polymer brush nanocomposite.

Based on the presence of various functional groups in the catalyst components, FTIR spectroscopy was used to characterize the content of the hybrid composite systems at various stages of the synthetic preparation (Figure 2). In Figure 2, the spectrum of the PAAgCHI/Fe_3_O_4_ hybrid particles display strong IR bands at 580 cm^−1^ and 3410 cm^−1^, which are characteristic of the Fe-O and OH stretching vibrational bands [29]. The IR bands observed near 1600 cm^−1^ are attributed to the C=O and COO^−^ moieties of the carbonyl and carboxylate groups of the grafted PAA arms. After the reduction of Ag ions and incorporation of NPs onto the polymer brush, the FTIR spectra of the polymer brushes were significantly changed, and characteristic peaks for the PAAgCHI/Fe_3_O_4_/Ag samples appeared. The peaks for symmetric (1412 cm^−1^) and asymmetric (1580 cm^−1^) vibrations originating from carboxylate groups are shifted to lower wavenumbers, which confirms the reduction and assembly of Ag ions onto the carboxylate coordination sites (Figure 2b).

**Figure 3:**
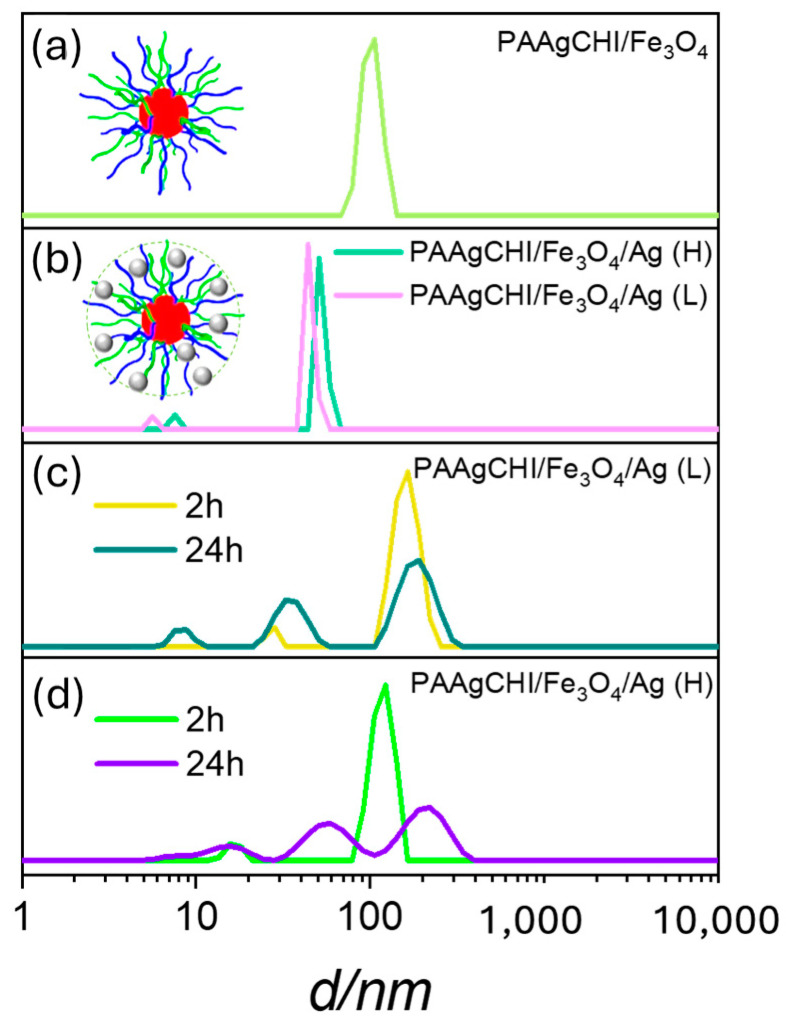
(**a**,**b**) Size distributions of materials obtained from DLS analysis for dilute aqueous solutions of PAAgCHI/Fe_3_O_4_, PAAgCHI/Fe_3_O_4_/Ag (H) and PAAgCHI/Fe_3_O_4_/Ag (L) at ambient pH, and (**c**,**d**) show nanocatalyst stability against aggregation in aqueous media for 24 h.

**Figure 4:**
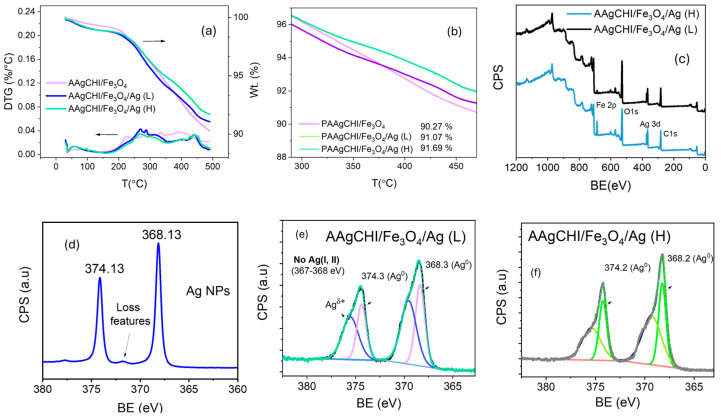
(**a**,**b**) TGA and derivative thermal gravimetric (DTG) of PAAgCHI/Fe_3_O_4_ and PAAgCHI/Fe_3_O_4_/Ag (H, L) nanocatalysts. (**c**) XPS survey of PAAgCHI/Fe_3_O_4_/Ag nanocatalysts. (**d**) Ag 3d peaks in *as-prepared* Ag nanoparticles, and (**e**,**f**) Ag 3d peaks in PAAgCHI/Fe_3_O_4_/Ag (H, L) nanocomposites.

The hydration and swelling behavior of the PAAgCHI/Fe_3_O_4_ and PAAgCHI/Fe_3_O_4_/Ag (H, L) catalyst systems after each functionalization step was evaluated using dynamic light scattering (DLS) measurements (Figure 3a,b). DLS results for the PAAgCHI/Fe_3_O_4_ and PAAgCHI/Fe_3_O_4_/Ag (H, L) catalyst systems revealed that the PAAgCHI polymer is fully swollen at ambient pH, which prevents the aggregation of the catalyst systems and AgNPs due to electrostatic and steric effects [30,31]. The hydrodynamic diameter (*D_h_*) of PAAgCHI/Fe_3_O_4_ is ca. 100 nm (Figure 3a). Interestingly, the PAAgCHI/Fe_3_O_4_/Ag (H, L) hybrid systems had markedly smaller hydrodynamic diameters than PAAgCHI/Fe_3_O_4_. The colloidal PAAgCHI/Fe_3_O_4_/Ag (H, L) hybrids had a diameter of ca. 70 nm upon attachment of AgNPs to the active sites within the magnetite/brush system (Figure 3b) [29,32]. The most probable scenario to account for this behavior is that the AgNPs incorporated into the magnetite brushes can act as cross-linkers between PAA and the magnetite nanocomposites and decrease their free movement and, consequently, reduce the hydration properties of the polyelectrolytes [22,29]. Furthermore, AgNPs can also decrease the hydrodynamic volume by weakening the hydration capabilities of polymer brushes due to a reduction in the effective number of -COOH groups and reduction in charge density within polyelectrolytes [22]. Finally, in order to further examine the colloidal stability of the materials, their behavior in an aqueous solution was monitored for 2 h and 24 h, where the results are shown in Figure 4c,d. After 2 h, the DLS measurements were performed, and the materials still showed no notable levels of aggregation in accordance with their colloidal stability and low likelihood of aggregation in aqueous media. Furthermore, the samples were kept undisturbed for an additional 24 h, where the estimated particle size from DLS was very similar to the original samples. A slight size increase was observed for the particles as the hydrodynamic diameter peaks shifted slightly to larger size distributions. The emergence of new scattering peaks that correspond to small size distributions centered at 30 and 20 nm was observed. This effect may relate to the presence of Ag or magnetite nanoparticles present in the solution. These findings indicate that this magnetic polymer brush is an effective support to stabilize and enhance the catalytic activity of AgNPs.

Thermogravimetric analysis (TGA) was performed to examine the weight content of the AgNPs within each polymer brush NC, where the results are presented in Figure 4a,b and Table 1. The TGA profile of the magnetite polymer brush without silver demonstrates a final weight of 90.3% at the temperature range of 25–500 °C, indicating that the grafting amount of polymer brush on magnetite was approximately 10% of the total mass. In comparison, the Ag-loaded catalysts show slightly higher metal residue than the previous sample, with final weights of 91.1% and 91.7% for PAAgCHI/Fe_3_O_4_/Ag (L) and PAAgCHI/Fe_3_O_4_/Ag (H), respectively. In turn, the TGA results were used to obtain the Ag concentration in the PAAgCHI/Fe_3_O_4_/Ag nanocatalysts. Considering the fact that the wt.% of PAAgCHI/Fe_3_O_4_ in both catalysts is the same, the concentrations of Ag in PAAgCHI/Fe_3_O_4_/Ag (H) and PAAgCHI/Fe_3_O_4_/Ag (L) were calculated as 1.4% and 0.8%, which are presented in Table 1. The AgNP wt.% is in line with the experimental procedure (Figure 4b) and concurs with the anticipated binding affinity between the Ag species and the carboxylate arms of PAA [24]. To further identify the formation and chemical bonding of Ag nanoparticles, the PAAgCHI/Fe_3_O_4_/Ag nanocatalysts were analyzed by XPS (Figure 4c–f). Figure 4c shows a wide spectrum of PAAgCHI/Fe_3_O_4_/Ag nanocatalysts, which clearly reveal the presence of Fe, O, C and Ag in the nanocatalysts. The XPS bands of 3d Ag in pristine AgNPs appear at binding energies of 374.13 and 368.13 eV as two doublets (Figure 4d) that correspond to metallic silver [33,34]. Compared to metallic AgNPs, the Ag 3d spectra in the PAAgCHI/Fe_3_O_4_/Ag nanocatalysts can be resolved into two species, which further confirms the successful embedding of AgNPs onto the magnetic polymer brushes (Figure 4e,f) [20,35].

### 3.2. Applications of Magnetic Nanocomposites in Heterogeneous Catalysis

#### 3.2.1. Catalytic Reduction of 4-Nitrophenol Using PAAgCHI/Fe_3_O_4_/Ag (H, L)

**Figure 5:**
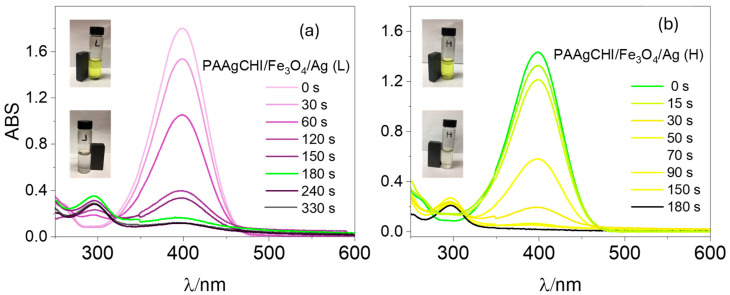
UV–vis spectra recorded during catalytic reduction of 4-nitrophenol to 4-aminophenol at different times for (**a**) PAAgCHI/Fe_3_O_4_/Ag (H) and (**b**) PAAgCHI/Fe_3_O_4_/Ag (L) nanocatalysts in the presence of NaBH_4_. Conditions: 4-nitrophenol solution; 5 mL, 1.1 mM. Catalyst dosage; 5 mg and NaBH_4_ dosage; 10 mg.

To examine the potential applications of the polymer brush/Ag nanocomposites as catalysts, two types of catalysts, namely PAAgCHI/Fe_3_O_4_/Ag (H, L), were tested in the catalytic reduction of methylene blue and methyl orange dyes in the presence of NaBH_4_. The following two representative organic dyes (4-nitrophenol and methyl orange) possess mutagenic and carcinogenic properties [36,37], where the removal of such dyes is of key importance in water decontamination processes [38,39],. For comparison, the catalytic activity of pure AgNPs and the PAAgCHI/Fe_3_O_4_ composite was evaluated. However, for both dyes, these materials displayed comparatively low or poor catalytic activity overall for dye reduction [15]. The reduction in catalytic activity was monitored via UV–vis spectrophotometry with a spectral range of 200–600 nm for monitoring the dye absorbance of 4-NP at 400 nm at variable times. It was observed that any negligible reaction occurred in the absence of any catalyst [12]. The reduction of 4-NP in the sole presence of AgNPs (without catalyst support) required a longer time of approximately 50 min (Figure 2a, reference [12]). While a similar reaction, using polymer-supported AgNPs as catalysts, showed an enhanced reaction rate, and the reduction was completed within 180 s and 330 s for PAAgCHI/Fe_3_O_4_/Ag (H) and PAAgCHI/Fe_3_O_4_/Ag (L) NCs, respectively (Figure 5a,b). The color of the 4-NP solution changed from yellow to colorless as the reduction proceeded, which is shown by photographic images in the insets of Figure 5a,b.

**Figure 6:**
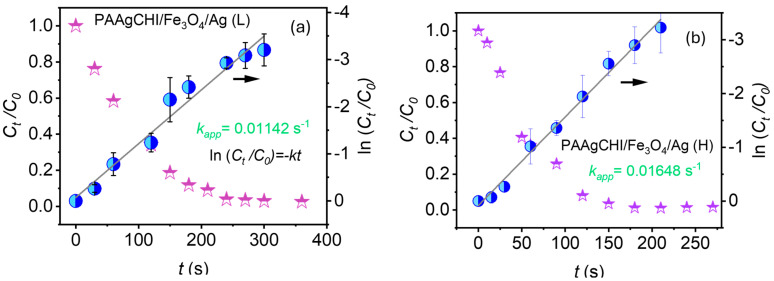
Plotted kinetic data for (**a**) PAAgCHI/Fe_3_O_4_/Ag (L) and (**b**) PAAgCHI/Fe_3_O_4_/Ag (H) for the reduction of 4-nitrophenol.

For the kinetics experiments, NaBH_4_ was added in excess relative to 4-NP, which allows to safely assume that the rate constant (κ) of the catalytic reduction is independent of the NaBH_4_ concentration. Therefore, the reduction reaction was observed to follow first-order kinetics with regard to 4-NP [40,41]. Figure 6a,b show the variation in the ratio of 4-NP absorbance (Abs) vs. time (*t*) to its initial Abs, (*C_t_/C*_0_) as a function of time for both PAAgCHI/Fe_3_O_4_/Ag (H, L) catalysts. According to the Beer–Lambert law, absorbance is proportional to concentration; thus, the values *C_t_* and *C*_0_ represent Abs of 4-NP in the system (the intensity of the characteristic band at 400 nm) at variable time intervals and at t = zero, respectively. The regression plot of *ln*(*C_t_/C*_0_) versus reaction time showed a linear correlation for PAAgCHI/Fe_3_O_4_/Ag (H, L), as shown in Figure 6a,b. From the plot of kinetics, the values for the apparent rate constant were calculated from the respective slopes for each catalyst and were 0.016 s^−1^ (0.96 min^−1^) for PAAgCHI/Fe_3_O_4_/Ag (H) and 0.011 s^−1^ (0.66 min^−1^) for PAAgCHI/Fe_3_O_4_/Ag (L). By comparison, the slope value for the as-prepared AgNPs was 4.8 × 10^−4^ s^−1^ [15]. The superior performance with reference to AgNPs (ca. 33 times more active) indicates that the polymer brush contributes immensely to the overall catalytic efficiency.

The reaction efficiency of a catalyst is strongly affected by the possibility of contact between the catalytic active sites and the reactants. For the PAAgCHI/Fe3O4/Ag (H, L) nanocatalysts, the catalytic enhancement efficiency is attributed to a synergistic effect of the hydrophilic polymer brush and the magnetite NPs, which allows for uniform distribution of AgNPs onto the hydrophilic polyelectrolyte brush. This leads to increased contact opportunities between hydrophilic reactants and Ag active sites, where the 4-NP dye molecules can readily diffuse through the polymer brush to contact the surface of the silver NPs. Furthermore, we compared the catalytic activities (k_a_ = k/mass of catalyst) of the PAAgCHI/Fe_3_O_4_/Ag (H, L) materials to similar Ag nanocatalysts, as shown in Table 2. The results presented reveal that the catalytic activities observed for both nanocatalysts reported in this study outperformed their Ag-based counterparts from the literature by up to two orders of magnitude.

#### 3.2.2. Catalytic Reduction of Methyl Orange Using PAAgCHI/Fe_3_O_4_/Ag (H, L)

**Figure 7:**
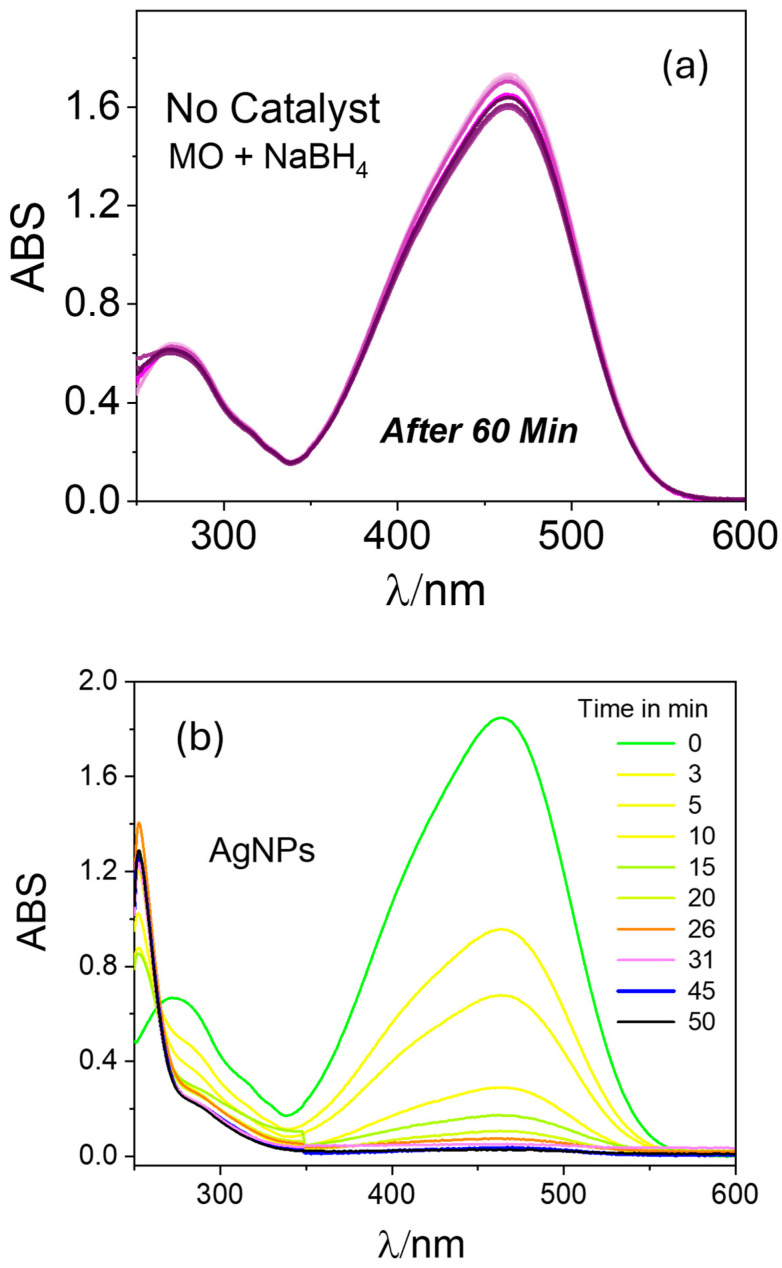
UV–vis spectra recorded during catalytic reduction of methyl orange at variable times and conditions: (**a**) no catalyst and (**b**) unsupported AgNPs catalyst were employed. Conditions: Methyl orange solution; 5 mL, 1.0 mM. Catalyst dosage; 5 mg. NaBH_4_ dosage; 10 mg.

**Figure 8:**
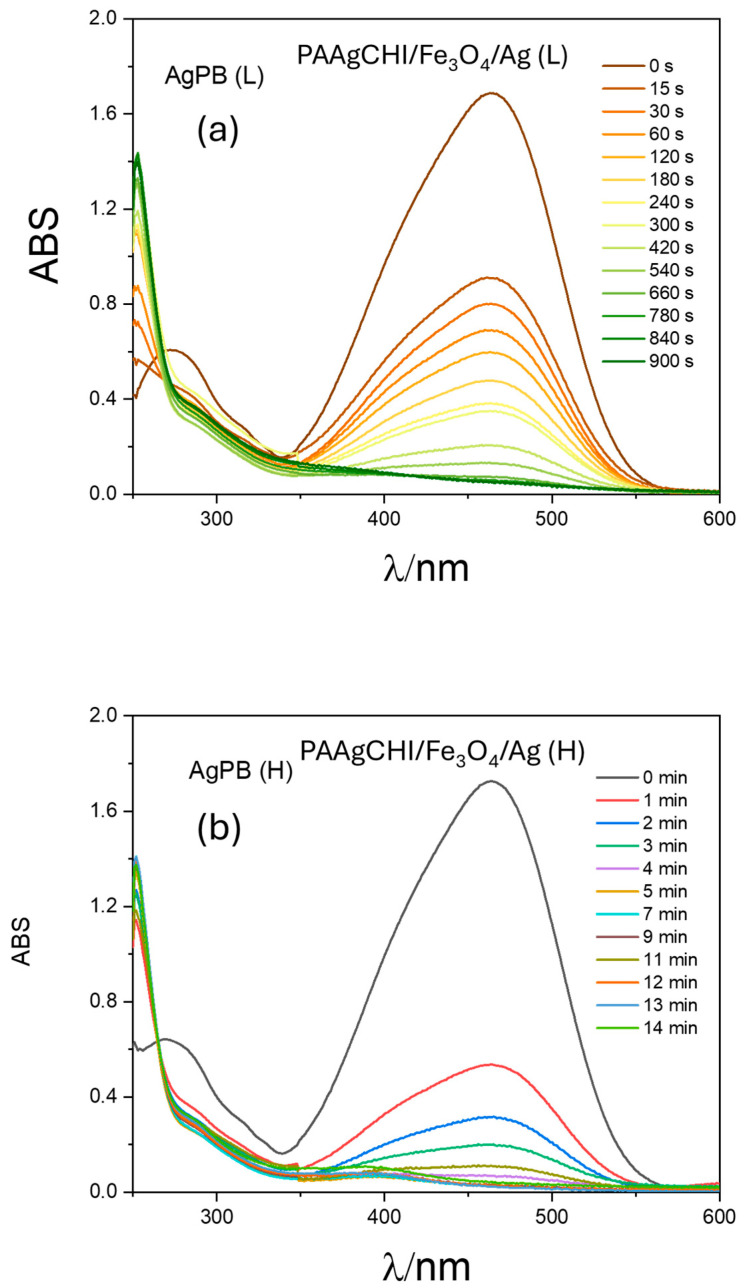
UV–vis spectra recorded during catalytic reduction of methyl orange at variable times and conditions: (**a**) PAAgCHI/Fe_3_O_4_/Ag (L) and (**b**) PAAgCHI/Fe_3_O_4_/Ag (H) catalysts were employed. Conditions: methyl orange solution; 5 mL, 1.0 mM. Catalyst dosage; 5 mg. NaBH_4_ dosage; 10 mg.

To verify the efficiency of the prepared catalysts for other dye species, we conducted similar catalytic reduction experiments for MO using the PAAgCHI/Fe_3_O_4_/Ag (H, L) catalyst NPs. The reaction progress was monitored using UV–vis spectrophotometry over a 250–600 nm spectral range according to the decay in the MO absorbance band at 470 nm [44,45,46]. Apparently, aqueous solutions of MO show an intense absorption band at 470 nm. In the absence of any catalyst, the reaction did not proceed as illustrated in Figure 7a. When the reaction was performed using as-prepared AgNPs as the catalyst, the reduction process progressed to completion in almost 50 min (Figure 7b), indicating the negligible catalytic activity of AgNPs for methyl orange reduction. By contrast, upon addition of the PAAgCHI/Fe_3_O_4_/Ag (H, L) nanocomposites into the solution, the catalytic reduction progressed very rapidly (ca. 3.3- to 3.6-fold rate enhancement), where the reduction required approximately 14 and 15 min for completion by the PAAgCHI/Fe_3_O_4_/Ag (H) and PAAgCHI/Fe_3_O_4_/Ag (L) catalyst systems. The obvious change in time-dependent absorption at 470 nm is illustrated in Figure 8a,b.

**Figure 9:**
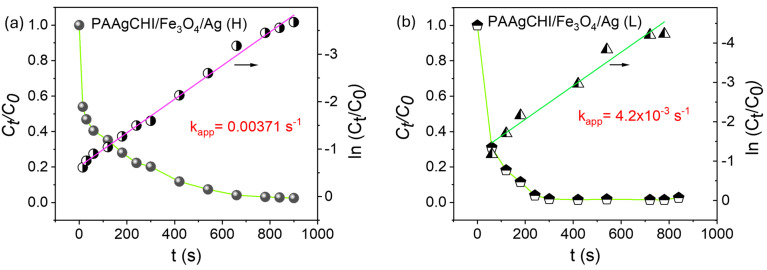
Kinetic profiles for the reduction of methyl orange: (**a**) PAAgCHI/Fe_3_O_4_/Ag (H) and (**b**) PAAgCHI/Fe_3_O_4_/Ag (L) nanocatalyst systems.

A similar procedure for 4-NP was followed to investigate the kinetics of reduction for MO using the PAAgCHI/Fe_3_O_4_/Ag (H, L) catalysts, where the results are presented in Figure 9a,b. A linear relationship of the regression plot of *ln*(*C_t_/C*_0_) versus reaction time showed a linear profile for both of the catalyst NCs (PAAgCHI/Fe_3_O_4_/Ag (H, L)), as illustrated in Figure 9a,b. The apparent rate constants of MO reduction were based on the resulting slopes, which were estimated as 0.0037 s^−1^ and 4.2 × 10^−3^ s^−1^ for the PAAgCHI/Fe_3_O_4_/Ag (H) and PAAgCHI/Fe_3_O_4_/Ag (L) nanocatalysts, respectively. The notable enhancement of the catalytic activity for the NCs versus the as-prepared AgNPs (ca. three-fold enhancement) indicates the potential applicability of these nanocatalysts for variable types of dye species.

**Figure 10:**
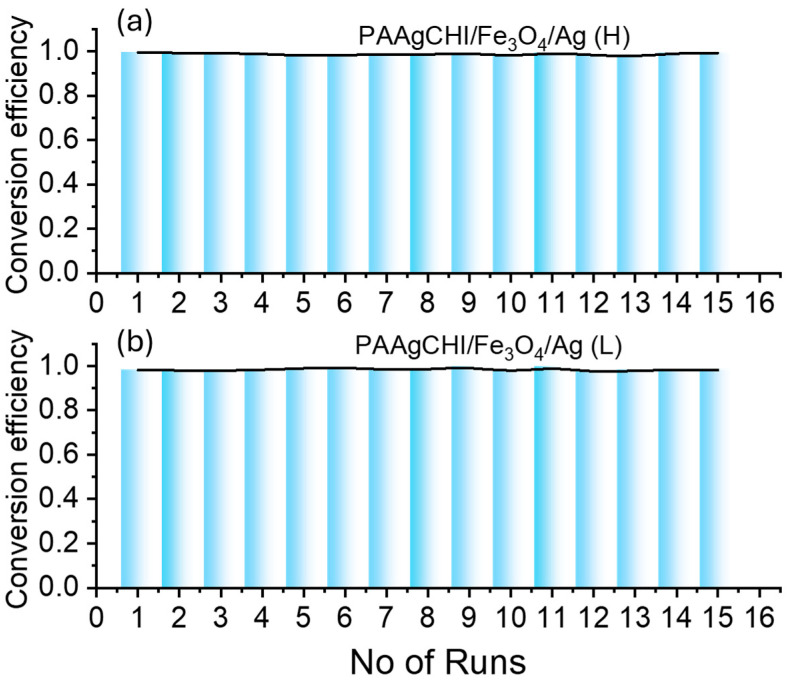
Reusability of the nanocatalysts over the fifteen consecutive catalytic cycles for reduction of 4-nitrophenol: (**a**) PAAgCHI/Fe_3_O_4_/Ag (H) and (**b**) PAAgCHI/Fe_3_O_4_/Ag (L) NCs.

The potential reuse of the catalyst particles was examined by performing a series of consecutive reduction reaction experiments after the completion of the first cycle of reduction. The experiments were performed by removal of materials from the solution using a magnet and adding into a fresh mixture of 4-NP to run the next cycle. Figure 10a,b show typical data for the reusability of the PAAgCHI/Fe_3_O_4_/Ag (H, L) nanocomposites for 15 cycles. It was observed that the catalytic activity of the NCs was preserved, as demonstrated by an excellent conversion efficiency (~99–98%) that was maintained for both types of catalyst systems.

## 4. Conclusions

Uniformly dispersed and catalytically active Ag nanocomposites (PAAgCHI/Fe_3_O_4_/Ag (H, L)) that consist of magnetic PAAgCHI/Fe_3_O_4_ brushes were prepared by stabilizing Ag nanoparticles within an anionic polyelectrolyte biopolymer grafted brush system at two different loading levels of AgNPs. The resulting hybrid materials showed excellent catalytic activity with reference to as-prepared silver NPs for the reduction of methyl orange and 4-nitrophenol dyes. The PAAgCHI/Fe_3_O_4_/Ag (H, L) materials also showed excellent colloidal stability that results from the electrostatic repulsion between adjacent polyelectrolyte polymer brushes, which offered more rapid reduction kinetics. Furthermore, the catalyst particles possess a high degree of recyclability over multiple cycles (up to *n* = 15). This strategy of conjugating AgNPs onto stimuli-responsive magnetic polyelectrolyte brushes yields well-dispersed and magnetically retrievable nanocatalysts with excellent performance over pristine metal nanoparticles. The enhanced sustainability of the nanocatalysts reported in this study offers key advantages for the reduction of organic dyes in advanced water treatment and environmental remediation.

## Figures and Tables

**Table 1:** Summary of TGA results for magnetite polymer brush and silver loaded nanocomposites.

Material	Total Weight Loss (%)	Calculated Weight % of Silver
PAAgCHI/Fe_3_O_4_	9.73	0.0
PAAgCHI/Fe_3_O_4_/Ag (L)	8.93	0.8
PAAgCHI/Fe_3_O_4_/Ag (H)	8.31	1.4

**Table 2:** Comparison of the catalytic activities of PAAgCHI/Fe_3_O_4_/Ag (H, L) for the reduction of 4-nitrophenol with other known Ag catalyst materials.

Sample	Catalytic Activity *k_a_* (min^−1^ g^−1^)	Reference
PAAgCHI/Fe_3_O_4_/Ag (H)	96	This work
PAAgCHI/Fe_3_O_4_/Ag (L)	66	This work
Fe_3_O_4_@SiO_2_-Ag	0.46	Chi et al. [41]
SiO_2_@AgNPs	14.9	Wang et al. [42]
Ag@egg shell membrane	0.41	Liang et al. [43]

## Data Availability

The raw data supporting the conclusions of this article will be made available by the authors on request.
